# Cholangiokines: undervalued modulators in the hepatic microenvironment

**DOI:** 10.3389/fimmu.2023.1192840

**Published:** 2023-05-16

**Authors:** Xiurong Cai, Frank Tacke, Adrien Guillot, Hanyang Liu

**Affiliations:** ^1^ Department of Hematology, Oncology and Tumor Immunology, Charité Universitätsmedizin Berlin, Campus Virchow-Klinikum, Berlin, Germany; ^2^ Department of Hepatology and Gastroenterology, Charité Universitätsmedizin Berlin, Campus Virchow-Klinikum and Campus Charité Mitte, Berlin, Germany; ^3^ Center of Gastrointestinal Diseases, Changzhou Second People’s Hospital, Changzhou Medical Center, Nanjing Medical University, Changzhou, China

**Keywords:** biliary epithelial cells, cholangiocyte secretome, cholangiopathies, ductular reaction, cellular senescence, inflammation, fibrosis, hepatic carcinogenesis

## Abstract

The biliary epithelial cells, also known as cholangiocytes, line the intra- and extrahepatic bile ducts, forming a barrier between intra- and extra-ductal environments. Cholangiocytes are mostly known to modulate bile composition and transportation. In hepatobiliary diseases, bile duct injury leads to drastic alterations in cholangiocyte phenotypes and their release of soluble mediators, which can vary depending on the original insult and cellular states (quiescence, senescence, or proliferation). The cholangiocyte-secreted cytokines (also termed cholangiokines) drive ductular cell proliferation, portal inflammation and fibrosis, and carcinogenesis. Hence, despite the previous consensus that cholangiocytes are bystanders in liver diseases, their diverse secretome plays critical roles in modulating the intrahepatic microenvironment. This review summarizes recent insights into the cholangiokines under both physiological and pathological conditions, especially as they occur during liver injury-regeneration, inflammation, fibrosis and malignant transformation processes.

## Introduction

Cholangiocytes, also known as biliary epithelial cells (BECs), are specialized epithelial cells forming the biliary epithelium and lining the bile ducts (1). In general, cholangiocytes are polarized with apical and basal membranes corresponding to different functions: 1) maintain bile flow *via* the cilium system and intraductal homeostasis *via* active biomolecule transport; 2) modify bile *via* secreting bicarbonate (HCO_3_^−^) through the plasma membrane domain; 3) maintain cross-ductal interaction in the liver, depending on their tight junctions and immunoglobulin A (IgA) secretion; 4) reabsorb different molecules, including bile salts, bile acids, glucose, amino acids and ions (2–4). Cholangiocytes represent a heterogeneous population in terms of morphological characteristics, classically described as small or large cholangiocytes (5). Accordingly, cholangiocyte transcriptome is highly variable, so as their structural and metabolic functions. Large cholangiocytes typically line the larger branches of the biliary tree and form more complex structures than those small cholangiocytes. Simultaneously, large cholangiocytes engage in hormone-modulated bile secretion, while small cholangiocytes are able to proliferate and exhibit functional plasticity in diseases (6–8). Small cholangiocytes appear more capable of self-replication during liver injury, implying their potential in the liver regeneration and ductular reaction (DR) (9). DR is described as a complex of dynamic interactions among liver parenchymal cells, stromal cells, and immune cells, which serves a crucial machinery during liver injury-regeneration, fibrogenesis, and malignant transformation processes. Though not affirmatively being recognized as the origins of DR, cholangiocytes participate in DR as both initiators and executors (10).

To date, cholangiocyte biology has been merely studied in liver diseases, due to their relatively small population in the liver. However, a rising number of studies unveiled crucial functions of cholangiocytes in liver pathobiology. Interacting with both intra- and extrahepatic ductal environments, cholangiocytes are exposed to both hepatic molecules and gut-derived stimuli [pathogen-associated molecular patterns (PAMPs), danger-associated molecular patterns (DAMPs) and microorganisms] (11, 12). Cholangiocytes have been identified as collateral targets of various liver diseases such as fatty liver disease [nonalcoholic fatty liver disease (NAFLD)/non-alcoholic steatohepatitis (NASH)] and alcohol-related liver disease (ALD). BECs are also directly injured in chronic cholestatic liver diseases including primary biliary cholangitis (PBC), primary sclerosing cholangitis (PSC), biliary atresia (BA) and cholangiocarcinoma (13, 14). Furthermore, stimulated cholangiocytes can adopt varying secretory phenotypes. Importantly, bile duct-derived ductular cells are considered to play active roles in liver regeneration, although contradictory results suggest the necessity of a more comprehensive analysis of their function. More interestingly, activated cholangiocytes exhibit a peculiar secretory phenotype that dramatically shapes their surrounding microenvironment by modulating immune cell recruitment and mesenchymal cell migration and activation (15). From our current understanding, the release of cholangiokines (cholangiocyte-secreted cytokines, including chemokines, growth factors, ect.) is associated with cell statuses, which are affected by tissue inflammation, infection, and metabolic dysregulations. Both acute and chronic liver disorders have been shown to alter the BEC secretory profiles (16–18). Consequently, elevating attention has been given to cholangiokines in the liver, which inspires more work on obtaining in-depth and systematic understandings.

## Pathogenic triggers of cholangiocyte activation

More than constituents of bile ducts, cholangiocytes play a critical role in maintaining liver homeostasis, which refers to the balance of various physiological processes in the liver. One of the essential functions of cholangiocytes is to regulate biliary composition and bile flow by secreting and absorbing electrolytes, water, and other solutes. Standing to reason, cholangiocytes are vulnerable targets in cholangiopathies, which is a complex umbrella term encompassing inherited disorders, autoimmune or other poorly understood diseases (e.g., PSC, PBC and autoimmune cholangitis), exogenous stimuli-induced injury (e.g., infection and drug), ischemic injury and other undefined types of insults. Under such injury conditions, cholangiocytes assuasively secrete cholangiokines to sustain the microenvironment of the portal area. Moreover, cholangiocytes participate in the immune response and inflammation regulation through cytokine and chemokine production. Therefore, circulating immune cells are attracted and activated to promote portal inflammation (1, 19–22).

**Figure 1:**
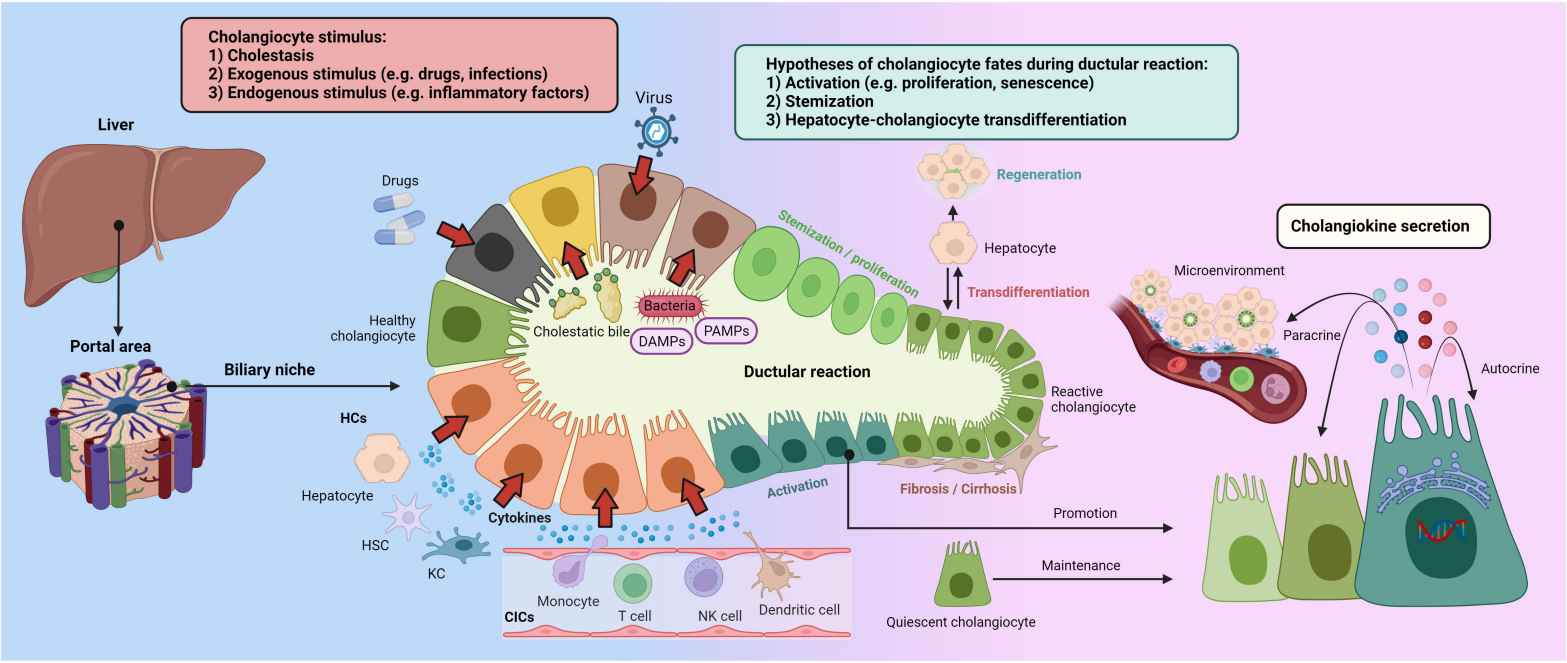
Ductular reaction and cholangiokine secretion Cholangiocytes stand as a crucial component in the hepatic portal areas, maintaining liver homeostasis. Cholangiocellular phenotypes can be altered by cholestasis, exogenous stimulus, and inflammatory factors. Besides evidence and debates on cholangiocyte stemization and hepatocyte-cholangiocyte trans-differentiation, activated cholangiocytes drive the DR by producing variable cytokines and chemokines, termed cholangiokines. Furthermore, cholangiokines are responsible for autocrine and paracrine effects in the portal microenvironment. CIC, circulating immune cell; DAMPs, damage-associated molecular patterns; HC, hepatic cell; HSC, hepatic stellate cell; KC, Kupffer cell; PAMPs, pathogen-associated molecular patterns.

According to variable chronic liver injury mouse models, BECs actively interact with hepatocytes and liver progenitor cells (HPCs) to promote the DR, which ultimately constitutes an alternative liver regeneration process (23, 24). Synchronously, BECs have been determined to fuel DR by several cholangiokines (25, 26). Hence, this section will demonstrate intriguing secretory phenotypes that occur in cholangiocytes, triggered by a complex portal niche (**Figure 1**).

### Cholestasis

Bile flow perturbation generally leads to an impaired bile efflux to the intestines, resulting in a pathogenic accumulation of bile acids in the intra-hepatic environment. Gradually concentrated and thickened bile exerts detrimental effects on the gut-liver axis, thus referred to as ‘toxic bile’ (27). Due to their anatomical location along the biliary tree, cholangiocytes are amongst the first cells to be affected by cholestasis. Studies have showed that higher levels of interleukin-8 (IL-8), a potent chemoattractant for neutrophils, were detected in the bile of PSC patients as compared to non-PSC patients (28, 29), which suggests that bile duct injury induces the secretion of inflammatory cytokines into the bile (30). Of note, the effects of the main bile salts, namely tauroursodeoxycholate (TUDC), taurocholate (TC), taurodeoxycholate (TDC), taurochenodeoxycholate (TCDC) and taurolithocholate (TLC), also remain to be defined. Results from Lamireau et al. indicated that TC effectively stimulated murine BECs to release monocyte chemoattractant protein-1/C-C motif chemokine ligand-2 (MCP-1/CCL-2) and IL-6 (31). In addition, oxysterols were revealed to insult cholangiocytes and induce malignancy transformation, which can be taken as a destructive part of ‘toxic bile’ (32–34).

Neuroendocrine hormones including secretin (Sct), are released by proliferating cholangiocytes (35, 36). Other than inducing biliary bicarbonate secretion by binding with its basolateral receptor (SR) (7, 37, 38), the Sct/SR axis plays a key role in the modulation of biliary proliferation and hepatic fibrosis by influencing the BEC secretome (35, 36, 39). The *SR* gene expression was shown to be elevated in the biliary obstruction animal model after bile duct ligation (BDL) (40). Furthermore, studies have shown that increased expression of vascular endothelial growth factor-A (VEGF-A) and transforming growth factor-beta 1 (TGF-β1) occurs when the Sct/SR axis is activated, leading to enhanced proliferation of ductular cells and fibrogenesis. Moreover, DR and liver fibrosis can be ameliorated when the SR expression was genetically disrupted in BDL and *Mdr2* ^−/−^ (*Abcb4* ^−/−^) mouse models (35, 39). Additionally, increased activity of the Sct/SR/TGF-β1 axis was observed in the liver of PSC patients compared to healthy livers (39).

Furthermore, fibroblast growth factor 19 (FGF19) was found in the liver samples from patients with cholestasis (41). Exposure to FGF19 has been associated with the proliferation and IL-6 release of cholangiocytes (42). More importantly, human gallbladder cells secrete FGF19 into the bile, which is assumed to participate in cholangiopathies (43). Even though many other cell populations have been identified as sources of FGF19, it would be interesting to elucidate the functions of cholangiocyte-secreted FGF19, particularly in the hepatoportal regions (44).

It has been reported that BDL-induced bile duct obstruction in mice triggers cholangiocytes to secrete osteopontin (OPN) (45, 46). Additionally, nerve growth factor (NGF) was found to be secreted by cholangiocytes in an experimental mouse cholestasis model (47). TGR5, well-known as a G-protein-coupled bile acid receptor, is highly expressed on cholangiocytes and hepatic macrophages. It is postulated that TGR5 can participate in bile production, proliferation regulation and inflammation modulation. The beneficial secretion of bicarbonate and chloride was known attributing to the TGR5-mediated cholangiocyte activation (48). Furthermore, it is hypothesized that TGR5 might impede hepatic cell-cell communication, which either directly or indirectly affects the cholangiocyte-associated secretory characteristics. Nonetheless, the effects of TGR5 on the cholangiocyte secretome have already been discussed elsewhere (49).

### Exogenous stimulus

Environmental factors (microorganisms, drugs, ischemia, etc.) serve a pivotal role in the cholangiocyte activation and pathogenesis of cholangiopathies. Notably, the microbiota has emerged as a crucial mediator of BEC functions (50). Although hepatocytes and Kupffer cells are generally responsible for the clearance of bacterial products in the liver (51, 52), cholangiocytes can also play auxiliary roles in this process, especially regarding the response to intraductal stimuli. In terms of physiology, the gut barrier stands as the first line of defense preventing external insults from entering the organism *via* the venous system, while bile duct acts as the front-line defending bile-derived insults (12). The portal channel, however, may allow certain bacteria, PAMPs, and DAMPs to enter the liver, affecting biliary inflammation or possibly inducing inflammation in the biliary tree. Paik et al. recently reported that gut-resident bacteria can inhibit Th-17 cell functions by producing bile acids (3-oxoLCA), which evidences a bacteria-induced immune turbulencein the gut-liver inflammatory modulation (53). Additionally, a growing number of studies have revealed the critical functions that gut microbiota plays in influencing the progression of liver disease, particularly in PSC and PBC (54–56). Indeed, PAMPs refluxed into the bile duct can be sensed by cholangiocytes *via* pattern recognition receptors, which can provoke a variety of inflammatory signaling pathways and cytokine secretion.

Fundamentally, cholangiocytes express the Toll-like receptor (TLR) family proteins, which are well-known as mediators in innate immune responses (57, 58). TLRs can recognize microbial and other exogenous molecules, PAMPs and DAMPs. Furthermore, the TLR activation induced by PAMPs and DAMPs triggering their conrrespondingsignaling pathways, results in the recruitment of toll/IL-1-domain containing adaptor molecules [e.g., myeloid differentiation protein 88 (MyD88)], and the activation of protein kinases [e.g., IL-1 receptor associated kinase (IRAK)]. The activation of these specific intracellular pathways leads to a nuclear factor kappa-B (NF-κB)-dependent secretion of proinflammatory cytokines/chemokines (59). Furthermore, cholangiocellular autocrine and paracrine signals are robustly enhanced by several cytokines including IL-1, IL-6, IL-8 and interferon-γ (IFN-γ) (60). Moreover, emerging studies have elucidated the mechanisms of microbiota implication on cholangiopathies. For instance, PBC patients were found to tolerate autoantibodies that can cross-react with bacterial antigens from *E. coli* and *N. aromaticivorans* (61). *E. coli* infection is known as a key factor in breaking immunological tolerance against the mitochondria, resulting in the production of PBC-specific autoantibodies (termed anti-mitochondrial autoantibodies) (62). These findings lend credence to prospective mechanisms underlying the secretome changes in association with cholangiocytes.

In the past three years, Coronavirus disease 2019 (COVID-19) has swept the world and brought new challenges to human diseases, leading to investigations and discussions on the COVID-19-interfered cholangiopathies (63, 64). A case report of COVID-19 patients discovered unique histologic features, including severe cholangiocyte injury and intrahepatic microangiopathy in their liver samples, suggesting a SARS-CoV-2-induced hepatic injury (64). Additionally, SARS-CoV-2 can infect cholangiocytes *via* the angiotensin-converting enzyme 2 (ACE2), which can be reduced by ursodeoxycholic acid while being induced by farnesoid X receptor (FXR) signaling in cholangiocytes (65).

Drug-induced cholangiopathies [also known as drug-induced vanishing bile duct syndrome (VBDS)] were first described in rare clinical cases (66). Certain medications, including carbamazepine and amoxicillin/clavulanic acid, have been shown to cause biliary damage (67). Additionally, fluorodeoxyuridines and 5-fluorouracil were revealed to selectively induce injuries in large bile ducts (68). Interestingly, cholangiocytes are implicated in drug metabolism as they were shown to express cytochrome P450 (CYP450) superfamily members (69, 70). Therefore, functional investigations linking drug metabolism or drug-induced liver damage to the secretory characteristics of cholangiocytes are highly anticipated.

Similar cholangiopathies with vanishing bile ducts, biliary strictures and protein casts also occur after ischemic insults, including ischemic-type biliary lesions (ITBL) after liver transplantation, secondary sclerosing cholangitis of critically ill patients (SC-CIP) after acute respiratory distress syndrome, COVID-19, shock and sepsis (71). Regrettably, most studies only investigated cellular injury or histological manifestations of such cholangiopathies without a detailed description of cholangiocyte-associated secretory phenotypes.

### Endogenous stimulus

In comparison to injuries, endogenous stimulus, mainly inflammatory factors, play pivotal roles in modulating a variety of cholangiocyte phenotypes. In this context, cholangiocytes act as a major sensor rather than an initiator of inflammation, which possibly explains the general notion of cholangiopathies in most acute and chronic liver diseases (14).

When there is a disturbance in homeostasis, cholangiocytes are more susceptible to immunological responses, which enhances their secretion of cytokines including chemokines, and angiogenic growth factors. For instance, IL-1 and tumor necrosis factor-α (TNF-α) trigger cholangiocytes to release epithelial cell-derived neutrophil-activating protein (ENA-78) and growth-related gene products (72). Moreover, primary human cholangiocytes treated with cytokines (IL-1β, TNF-α and IL-17) or TLRs-related PAMPs [Pam3CSK4, poly(I:C) and LPS] can attract periductal Langerhans cells (Langerin^+^ periductal cells) *via* secreting the chemokine macrophage inflammatory protein-3α (MIP-3α) to activate PAMPs-sensing TLRs, thereby regulating biliary innate immune response in PBC (73). Poly (I:C)-treated primary cholangiocytes, mimicking biliary damage in BA, also trigger a stronger release of chemokine (C-X3-C motif) ligand 1 (CX3CL1) and the subsequent attraction of malfunctional natural killer (NK) cells (74). Other cytokines (IL-1β, IL-6, and IL-23p19) and chemokines [chemokine (C-XC-C motif) ligand (CXCL)-1/2/3/6/8, CCL-2 and CCL-20) were found enriched in the interlobular bile ducts from PBC patients, which was also confirmed in the *in vitro* stimulation of primary cholangiocytes with PAMPs [Pam3CSK4, poly(I:C) and LPS] and IL-17 (75).

During persistent liver injury, cholangiocytes synthesize and release TGF-β, especially TGF-β2, which was significantly increased in reactive bile ducts of fibrotic livers. In turn, TGF-β further promotes cholangiocytes to secrete endothelin-1 and regulates, in a paracrine manner, the deposition of extracellular matrix in the adjacent mesenchymal cells (76). Cholangiocytes appear to be responsive to IFN-γ, is mainly secreted by CD8^+^ T cells (77). IFN-γ was revealed to ameliorate fibrosis and cholestasis in carbon tetrachloride-treated mice (78). On the other hand, IFN-γ induces a shift of cytokine secretion in cholangiocytes from an acute inflammation pattern to a chronic inflammation feature, serving an important driver of persistent inflammation in cholangiopathies. Specifically, IFN-γ represses IL-8 secretion while enhancing the secretion of several cytokines including MCP-1 (79), monokine (80), interferon-inducible T cell alpha chemoattractant (ITAC) (81) and interferon-γ-inducible protein 10 (IP10) (82). Furthermore, IFN-γ and IL-6 stimulate nitric oxide (NO) production in cholangiocytes by inducing nitric oxide synthase-2 (NOS-2) expression (83). Besides, BECs exposed to IFN-γ exhibit a phenotypic flip between the acute and chronic inflammatory processes in terms of their release of inflammatory components (84).

IL-6, HGF and epidermal growth factors (EGF) can promote the proliferation of cholangiocytes *in vitro*, while the secretion of IL-6 can be further enhanced by IL-1β and phorbol myristate acetate (85). Exogenous IL-6 addition can also rescue the activin-A-induced growth inhibition of primary cholangiocytes *in vitro* (86). With the assistance of NO, IL-6 is involved in the LPS-induced sepsis-related systemic inflammatory response and is one of the most powerful mitogens for cholangiocytes (87, 88). Moreover, LPS and IFN-activated liver-derived macrophages (LDM) express high level of CD154 (also known as CD40 ligand, CD40L), which triggers the CD40-dependent changes of secreting proinflammatory cytokines with increased IL-3, IL-12p70, IL-10 and GM-CSF but reduced CXCL10, IL-6 and CCL2 in human cholangiocytes (89). Studies have showed that TNF-α and IFN-γ could disrupt the barrier function of cholangiocytes (90–92). In addition, inflammatory macrophages secrete TNF-α in the earlier phases of liver diseases, causing an upregulation of integrin αvβ6 on the membrane of epithelial cells and leading to the binding and activation of latent TGF-β1 (93). Furthermore, BECs can produce MIP-3α/CCL-20 in response to cytokines (IL-1β, TNF-α and IL-17) and PAMPs (73). Such evidence suggests that mutual influence exists between macrophages and cholangiocytes during inflammatory hepatic processes.

Furthermore, the inflammatory milieu directly drives the alterations of the cholangiocyte secretory profile, leading to the recruitment of activated liver mesenchymal cells, thereby participating in the positive feedback loop of the inflammatory response as part of the DR. As a hallmark of epithelial–mesenchymal crosstalk, alterations in the reactive cholangiocyte secretome include the upregulation of TGF-β1, TGF-β2, IL-6, platelet-derived growth factor-B (PDGF-B) and CCL-2 (76, 94, 95). Hypothetically, endogenous stimulus derived by neighboring cells and circulating immune cells play constant roles in cholangiocyte activation and cholangiokine secretion, which eventually stimulate cholangiocytes to be a remarkable mediator in liver diseases.

## Cholangiocyte-associated secretory phenotypes

Under physiological conditions, cholangiocytes stay quiescent, maintaining both local and systemic bile homeostasis (38, 96). Even though the replication rate is limited in the quiescent state, cholangiocytes are able to re-enter cell-cycle and proliferate upon various exogenous or endogenous insults (97), and even compensate for the proliferation-incapable hepatocytes to regenerate liver parenchyma (98). The secretory dynamics of cholangiocytes act in autocrine, paracrine, and endocrine manners to maintain biliary homeostasis and regulate other cell types including hepatocytes, HSCs, portal fibroblasts (PFs) and immune cells (99). Cholangiocytes detect pathogens *via* the TLRs and then secrete antimicrobial IgA into the bile, which serves a vital barrier against germs from both the duodenum and portal vein (100, 101), as well as a variety of cytokines (IL-6 and MCP-1) (102), chemokines (103) and other active anti-microbial peptides (e.g., human beta-defensin-1, hBD-1) into the portal microenvironment (104). Accordingly, these secreted substances have a variable composition depending on the cellular state of cholangiocytes, which creates a complicated secretory network that distinguishes and maintains various cholangiopathies. In general, quiescent cholangiocytes in the biliary system become activated as a result of ongoing distress (e.g., targeted BEC injury and/or inflammatory response caused by broader liver insults). Active cholangiocytes have different cell cycle fates depending on the nature and duration of the injury, primarily cell death, growth and senescence (97).

Following an acute insult, injured cholangiocytes undergo cell death, either programmed (e.g., apoptosis) or non-programmed (e.g., necrosis). The release of apoptotic bodies or DAMPs can trigger a local inflammatory response, which aids in the clearance of cell debris, leading to a time-constrained immune response. However, when a moderate injury occurs or persists, cholangiocytes may re-enter the cell cycle, or engage into an irreversible cell cycle arrest (termed cellular senescence), both of which are accompanied by unique secretory patterns. Nevertheless, several factors, including IL-1β, IL-6, MCP-1, stem cell factor (SCF), TGF-β1, and PDGF, can be secreted by both proliferative and senescent cholangiocytes (105). The similarities and differences of secreted factors from cholangiocytes in proliferative and senescent states are described in the following sections. To understand the complexity of cell status-cholangiokine association, we summarize relevant evidence in **Table 1**.

**Table 1:** Cell status-associated cholangiokines.

Cell status	Cholangiokines	Conditions	Ref
Activated	MCP-1/CCL-2	Liver specimens from patients with chronic hepatitis	(106)
	CXCL-1/2/5/10/12, IL-1β and TGF-β1	CHF mouse model [Pkhd1(del4/del4]-deleted] derived primary cholangiocytes stimulated by CXCL-1 and -10	(93)
		Liver specimens from CHF patients	
	IL-8, TNF-α	PSC liver derived BECs exposed to TLR ligands (Pam3CSK4, LPS)	(107)
	IL-8	Human primary iBECs (from the non-neoplastic area of surgically resected livers of three patients with metastatic liver cancer) exposed to LPS and IL-1β and TNF-α	(108)
		Liver samples from patients with chronic viral hepatitis/liver cirrhosis/sepsis/extrahepatic biliary obstruction/fulminant hepatitis/PBC/PSC	
	Fractalkine	Human cholangiocarcinoma cell line (HuCC-T1) and human intrahepatic BEC line exposed to LPS and Th1-cytokines (IL-1β, IFN-γ and TNF-α)	(109)
Activated (proliferating)	IL-6	Human primary iBECs exposed to IL-1β and phorbol myristate acetate	(85)
	TGF-β2	Fibrotic specimens from patients with hepatitis B virus infection or alcohol abuse and rats with fibrosis secondary to bile duct ligation and scission.	(76)
	TGF-β1 and PDGF-BB	Mouse-derived iBEC organoids exposed to acetaminophen	(110)
Activated (injured)	IL-18	Mouse liver injury model (DDC diet) derived cholangiocytes exposed by LPS and ATP	(111)
		Liver samples from PSC patients	
	Fractalkine	Liver specimens from PBC patients	(109)
	CCL-2 and Integrin-β6	iBECs dissected from targeted biliary injury mouse model (*ihCD59^BEC-TG^*)	(112)
Activated (senescent)	TGF-β	Liver specimens from tamoxifen-inducible K19-Mdm2^flox/flox^ tdTom^LSL^ mice	(113)
	TGF-β1, MCP-1/CCL-2, IL-4, IL-5, IL-6, IL-7, IL-10 and IFN-γ	Liver specimens from PBC mouse model (dnTGF-βRII)	(114)
		Bile and liver specimens from PSC patients	
	CXCL-11, CCL-20	Serum from PBC patients	(115)
	CCL-2/3/4/5, CX3CL-1, CXCL-1, CXCL-2, CXCL-10 and CXCL-16	Mouse iBECs exposed to H_2_O_2_ and etoposide	(116)
	IL-6, IL-8, MCP-1/CCL-2, PAI-1	Normal human BECs exposed to LPS	(117)
		Liver specimens from PSC patients	
	MCP-1/CCL-2, CCL20, IL-3, IL-11 and IL-15	Mouse iBECs exposed to glycochenodeoxycholic acid	(118)
	TNF-α, IL-1β and MCP-1/CCL-2	Liver specimens from Mdr2^-/-^ mouse model	(119)
Quiescent	hBD-1	Human normal liver tissues	(104)
	Mucins and TFF	Human normal liver tissues	(120)
	Lactoferrin and Lysozyme	Human normal liver tissues	(121)
	Cathelicidin	Human normal liver tissues	(122)
	TGF-β2	Human normal liver tissues	(76)
	CCL-2, IL-8 and IL-4	Primary iBECs from the non-cancerous liver specimens of one iCCA patient	(102)
	MCP-1/CCL-2	Human normal liver tissue	(106)
	IGF-1	Rat normal liver tissues	(123)
		Liver samples from PBC patients	

CHF, congenital hepatic fibrosis; DDC, 3,5-diethoxycarbonyl-1,4-dihydrocollidine; iCCA, intrahepatic cholangiocarcinoma.

### Quiescence-associated secretory phenotypes

The anatomic location of the biliary system makes biliary epithelium a fundamental barrier against microorganisms mainly ascending from the duodenum and partially from the portal vein, or as suggested by recent studies present in the bile (1, 124–126). Thereby, under physiological conditions, cholangiocytes establish an intricate cooperative machinery with other hepatocytes and resident immune cells through direct or paracrine factor-mediated intercellular communication. This is supported by a recent study using single-cell RNA-sequencing data of human liver samples, revealing an up-regulation of genes involved in the secretion- and inflammation-related pathway in a subset of cholangiocytes, thereby indicating a crucial role of quiescent cholangiocyte secretome in maintaining homeostatic liver-biliary microenvironment. Additionally, it shows the heterogeneity of quiescent cholangiocyte populations in the liver, suggesting that cholangiocytes may be in a dynamic physiological state as they respond to occasional microbial assaults (127).

The majority of the immunoglobulins (Igs) in human bile are secretory Igs, which significantly maintain liver homeostasis. Hepatocytes effectively secrete most of the IgA in rodents, whereas cholangiocytes represent the main source of IgA secretion in human liver (101). Biliary immunoglobulins, especially IgA, are crucial innate defenders against microorganisms in the biliary tract and upper intestine. Quiescent cholangiocytes also secrete alternative antimicrobial peptides [such as defensins (104), mucins and mucin-associated trefoil peptides (TFF) (120), lactoferrin (121) and cathelicidin (122)], contributing to the basic defense of microorganisms in the biliary tract (128).

In addition to direct immunological defense through the bile, quiescent cholangiocytes can recruit and/or maintain different immune cells by expressing immune-modulating proteins on their surface or by secreting chemokines and cytokines (2). For instance, the cholangiocytes’ surface protein CD1d, which resembles the MHC class I molecule, can activate NKT cells by presenting lipid antigens (129). Cholangiocytes can also activate mucosal-associated invariant T (MAIT) cells, which are prevalent in the human liver and locate near bile ducts, by presenting bacterial antigens *via* MHC class I-related protein (130). Under normal circumstances, quiescent cholangiocytes secrete TGF-β2, which is involved in maintaining periductular connective tissues and is markedly up-regulated in the proliferating bile ducts of fibrotic livers (76). Last but not least, unstimulated primary human intrahepatic BECs secrete a panel of cytokines/chemokines *in vitro*, including IL-8, IL-4 and MCP-1 (102, 106), as well as insulin-like growth factor-1 (IGF-1) from healthy cholangiocytes (123).

Conclusively, in the healthy scenario, cholangiocytes mostly remain quiescent but retain baseline secretion of immunoglobulins, antimicrobial peptides, TGF-β2, IGF-1, etc., thereby dynamically maintaining the homeostatic hepatic microenvironment.

### Proliferation-associated secretory phenotypes

When a moderate injury occurs, cholangiocytes can re-proliferate to compensate for cell loss and repair the injury, which is aided by the acute inflammatory response. Cholangiocellular proliferation can be triggered by multiple pathways and stimulus, including IL-6, hepatocyte growth factor (HGF), estrogen, acetylcholine, and bile acids, all of which function through binding to their specific receptors (8). One fundamental feature of proliferating cholangiocytes is their enhanced secretion of variable pro-inflammatory cytokines, chemokines, growth factors, defensin, and other bioactive factors (99). With the timely repair of injury, inflammation would also resolve, and this scenario represents an acute inflammatory response without inducing aberrant hyperproliferation of cholangiocytes (131). However, if the damage persists to prevails over repair processes, cholangiocytes abnormally proliferate and induce chronic inflammation through interaction with various infiltrated immune cells, causing angiogenesis and fibrotic response in the liver, termed DR (14).

In response to a variety of insults, including infections, cholestasis and ischemia, quiescent cholangiocytes can be activated (97), and acquire a hyperproliferative and neuroendocrine-like phenotype with pro-fibrotic and pro-inflammatory secretome (57). Acting in an autocrine/paracrine fashion, these released bioactive factors, including pro-inflammatory cytokines and chemokines (e.g., IL-6, IL-8, TNF-α and various growth factors), modulate cholangiocyte biology and direct the prognosis of biliary damage (108, 132–134). For example, IL-8 and TNF-α levels are substantially elevated in cholangiocytes from individuals with advanced PSC compared to those at the early disease stage (107). In the infection scenario, *Helicobacter bilis* or fluke products (Opisthorchis viverrini excretory/secretory products) can activate cholangiocytes to proliferate and massively secrete IL-6 and IL-8, thereby initiating innate mucosal immunity against microorganisms (135, 136).

Other chemokines secreted by reactive proliferating cholangiocytes including fractalkine from injured small bile ducts of PBC. Fractalkine possesses chemoattractant and cell-adhesive functions in recruiting intraepithelial monocytes/lymphocytes by binding to its receptor CX3CR1 (109). Moreover, MCP-1 expression was intensively but not exclusively up-regulated in the epithelial cells of regenerating bile ducts (106), which contributes to myofibroblastic trans-differentiation of portal fibroblasts, resulting in biliary fibrosis and cirrhosis (94).

### Senescence-associated secretory phenotypes

In a chronic damage scenario or under a susceptible genetic background, injury-induced inflammation persists and causes cellular senescence in the biliary epithelium. Senescence can be induced by various factors, including repetitive replication-related telomere shortening, oncogene activation or inactivation of tumor suppressor genes, DNA-damaging interventions, and oxygen radicals (137). The first unveiled feature of senescence is the irreversible cell cycle arrest, leading to the limitation of cell division *in vitro* (138). With the deepened investigation of senescence in organisms, more hallmarks of senescence have been revealed, including intracellular accumulation of dysfunctional mitochondria, epigenetic alteration, apoptosis resistance, metabolism changes and secretion of multiple bioactive factors, so-called senescence-associated secretory phenotypes (SASP) (139). The initiation of senescence is triggered by DNA damage response (DDR), resulting in the activation of the p53 and the ERK/ETS1/2 pathways, which ultimately up-regulate the expression of *p21^CIP1^* (also known as *CDKN1A*) and *p16^INK4a^* (also known as *CDKN2A*), respectively (140, 141). As cell cycle blockers, the overexpressed *p21^CIP1^* and *p16^INK4a^* prevent cells from entering S phase from the G1 phase. Moreover, unsolvable DDR activates the retinoblastoma (Rb) and p53 pathways and promotes the formation of promyelocytic leukemia nuclear bodies, which ultimately leads to senescence-associated heterochromatin foci (SAHF) through the ASF1A and HIRA chaperones (142, 143).

Senescent cholangiocytes are accumulated in patients with PSC and alcoholic steatohepatitis and are associated with disease exacerbation (117, 144, 145). Entering senescence enhances metabolic levels, allowing cholangiocytes to resist apoptosis. Furthermore, immune cells are responsible to eliminate these apoptosis-resistant cells to prevent abnormal growth and oncogenesis (142). Nonetheless, when senescent cells persist due to an unsuccessful immunologic clearance, these senescent cells may promote aggressiveness of their neighboring malignant cells (146) or even acquire a stem cell-like phenotype themselves once being released from cell cycle withdrawal (147). The significant role of senescent cells root from not only their cell-autonomous changes but also their non-cell autonomous traits for inducing neighboring cells into senescence through their secreted TGF-β, as a bystander effect (113). The bystander effect was also unveiled in the *in vitro N-Ras*-induced senescent cholangiocytes, which promoted cell cycle arrest and SASP secretion in their surrounding cholangiocytes (117).

Senescent cholangiocytes can be found in mouse PBC samples at the early disease stage, resulting from the over-activation of the Sct/SR pathway and its induced TGF-β1 secretion, which triggered cytokine-induced senescence in an autocrine manner. SASP from these senescent cholangiocytes activates Kupffer cells and HSCs in a paracrine manner, leading to local inflammation and liver fibrosis (114). Moreover, clinical evidence also indicated an unneglected role of SASP from cholangiocytes in various cholangiopathies. For instance, C-X-C motif chemokine ligand-11 (CXCL-11) and CCL-20 from senescent cholangiocytes showed predictive value in detecting ursodeoxycholic acid (UDCA) non-responsive PBC patients (115). Further *in vitro* study revealed that oxidative stress- and DNA damage-induced senescent BECs exhibited stronger secretion of chemokines (CCL-2/3/4/5, CX3CL-1, CXCL-1, CXCL-2, CXCL-10, and CXCL-16), thereby attracting monocyte/macrophage-like RAW264.7 cells, which suggested that the influence of senescent cholangiocytes on the pathogenesis of PBC was likely achieved by their environmental modulation (116). Furthermore, elevated secretion of pro-inflammatory factors [IL-6, IL-8, CCL-2, plasminogen activator inhibitor-1 (PAI-1)] was evident in senescent cholangiocytes in PSC (117). More evidence showed that CCL-2, CCL-20, IL-3, IL-11 and IL-15 were upregulated in senescent BECs as SASP (118).Even though senescent cholangiocytes are not well understood in BA, intrahepatic bile duct-derived organoids exhibited reduced cholangiocyte proliferation after receiving acetaminophen treatment, while enhancing the secretion of TGF-β1 and PDGF-BB, which indicated a possible role of senescence in this regard (110). The pro-inflammatory factors from SASP label senescent cholangiocytes as harmful actors involving in disease progression, which opens the door for senescence-targeted therapy, such as TGF-inhibition and senolytics, in the treatment of senescent cholangiocytes-related bile duct disorders. For example, genetic or pharmacological (Fisetin) elimination of cholangiocyte senescence reduced the release of inflammatory markers (TNF-α, IL-1 and MCP-1) and alleviated fibrosis in the progression of PSC (119).

Besides the canonical secretion of cholangiokines, cholangiocytes were reported to possibly release extracellular vesicles (EVs) containing IL-13Ra1 into the serum of PSC patients (148). Higher protein levels of Cystatin-S, IL-13Ra1, CD83, IL-1β and EMAP-2 were found in these serum EVs. However, whether and how these EVs are released by cholangiocytes are unclear due to the lack of EVs-tracing evidence. Furthermore, another study revealed that LPS-induced or PSC patient-derived senescent cholangiocytes can also release EVs, which contain multiple growth factors, including EGF, while containing low levels of cytokine/chemokine (149).

In summary, the secretory phenotypes of cholangiocytes are dynamically modified by intrinsic evolutionary factors during the life course of cholangiocytes, and by extrinsic microenvironmental factors engaging with cholangiocytes. Regarding the complexity of the cholangiocyte secretome, temporospatial regulation and cellular context must be taken into account when deciphering the role of cholangiocytes and other cell types in cholangiopathies.

## Influences of cholangiokines on the hepatic environment

**Figure 2:**
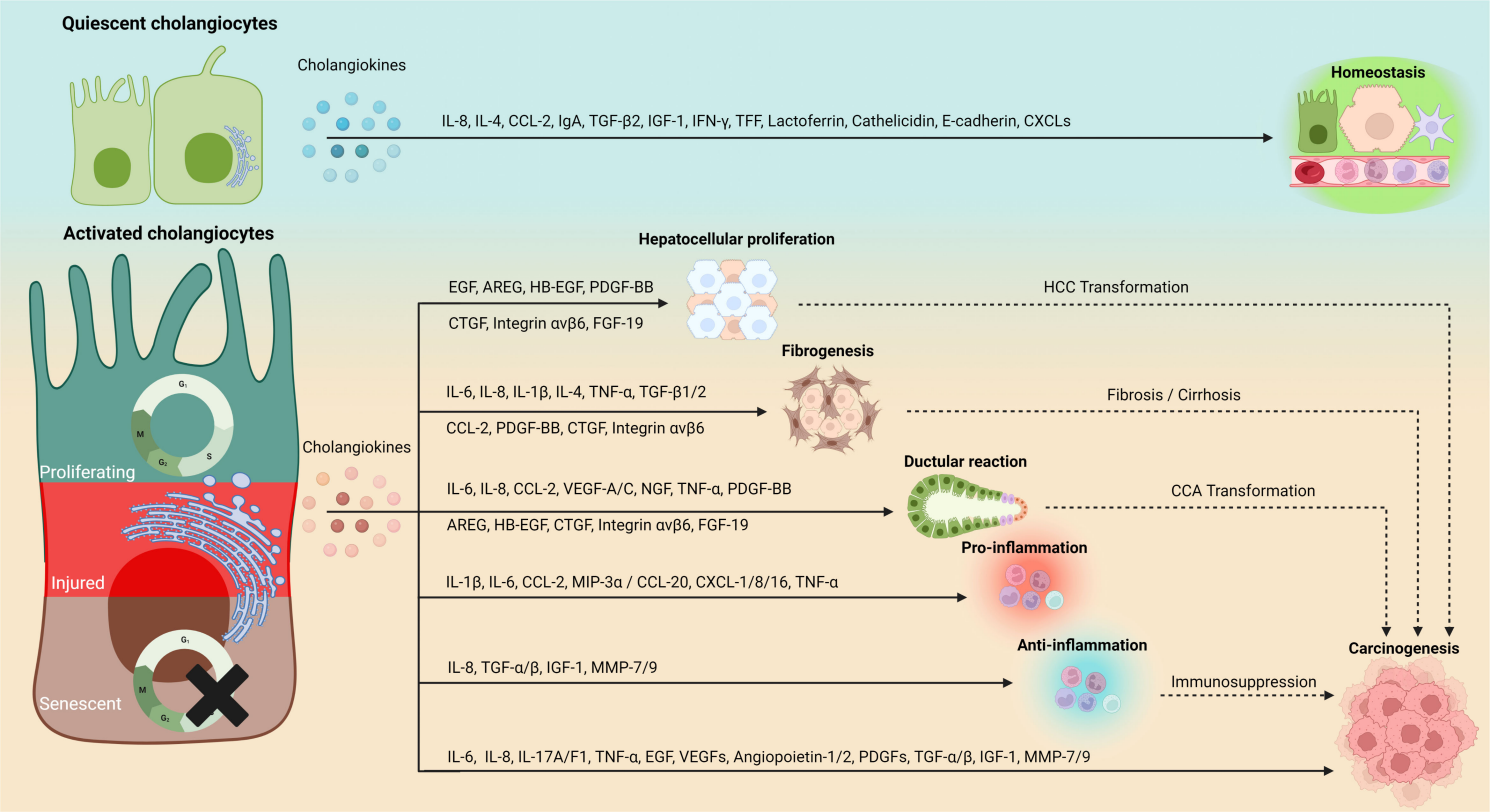
Phenotype-depending secretion and functionality of cholangiokines. Cholangiocytes convert to a major source of functional cytokines in addition to hepatocytes and immune cells. Cholangiokines exert perpetual influences on the hepatic environment. Quiescence-associated cholangiokines maintain liver homeostasis, whereas cholangiokines released by cholangiocytes at their activated statuses (e.g., proliferation, senescence and injury) mediate hepatocellular proliferation, fibrogenesis, DR and inflammation, which eventually cause hepatic carcinogenesis. IL, interleukin; CCL, chemokine (C-C motif) ligand; EGF, epidermal growth factor; IgA, immunoglobulin A; TGF-β, transforming growth factor-β; IGF-1, insulin-like growth factor 1; IFN-γ, Interferon gamma; TFF, trefoil factor; CXCL, chemokine (C-X-C motif) ligand; AREG, amphiregulin; HB-EGF, heparin-binding-EGF; FGF-19, fibroblast growth factor-19; TNF-α, tumor necrosis factor-α; VEGF, vascular endothelial growth factor, PDGF-BB, platelet-derived growth factor; CTGF, connective tissue growth factor; NGF, nerve growth factor; MIP-3α, macrophage inflammatory protein-3α; MMP, matrix metallopeptidase.

During liver injuries, biliary cells are susceptibly disturbed by both exogenous and endogenous stimulus, leading to cell damage. Thus, persistent damage and dysfunction in cholangiocytes trigger immune cell accumulation and inflammatory reaction, which cause variable pathological consequences, including excessive deposition of scar tissue in portal areas and biliary cirrhosis. This complex response triggered by immune cells, mesenchymal cells, and activated cholangiocytes is termed as DR (14). DR is orchestrated by a finely tuned interplay between proliferation, differentiation and trans-differentiation of cholangiocytes, hepatocytes and HPCs, ulteriorly fueling fibrogenesis and inflammation. Generally, in hepatobiliary diseases, DR refers to similar manifestations, including cholestasis, proliferation, inflammation, fibrosis, and eventually carcinogenesis (150). Nonetheless, the nature of DR remains obscure. As discussed in previous sections, cholangiocyte phenotype alterations (e.g., SASP, proliferation) during DR can drive cholangiocellular proliferation and inflammation by secreting cholangiokines, which further favors DR progression. Coinciding with current opinions, cholangiocytes are considered as not only reactors but also potential initiators in DR (14, 151, 152). In this context, cholangiokines may play crucial roles in different liver/bile duct pathological models by modulating complex cellular interactions, which will be discussed in detail in the following sections (**Figure 2**).

### Liver regeneration

The liver has a remarkable capacity to regenerate due to the persistent occurrence of hepatocyte self-renewal. While the facultative stemization of hepatocytes has been assumed as the main origin of liver regeneration for centuries, cholangiocyte proliferation and trans-differentiation appear to be a recently recognized mechanism to enhance the liver regenerative capacity (22).

Fundamentally, HGF and ligands of epidermal growth factor receptor (EGFR), viewed as ‘complete mitogens’, can induce hepatocyte proliferation, even in cultures without serum supplement (153). In terms of liver regeneration, EGF, amphiregulin (AREG), TGF-α and heparin-​binding EGF-​like growth factor (HB-​EGF) are more relevant to hepatocellular proliferation by binding to EGFR (22). Uriarte et al. discovered an increased secretion of HGF from HGF-19-treated murine cholangiocytes (154). In the Mdr2^-/-^ mouse model, senescent cholangiocytes were found enriched with multiple growth factors, including EGF (149). Zhao et al. used cholangiocytes with *Cul3* (known as a tumor suppressor) gene deficiency to show that cancerous cholangiocytes are prone to secrete AREG (155). Moreover, another study indicated that cancerous cholangiocytes upregulated HSC-based HB-​EGF upon TGF-β secretion (156). In addition, TNFs and IL-6 are known as ‘auxiliary mitogens’. A delayed liver regeneration was recorded in mice with genetic TNF receptor 1/2 (TNFR1/2)-deficiency (157) Simultaneously, IL-6-deficient mice showed reduced activation of hepatocellular STAT3, which is a determinant in promoting proliferation (158). Interestingly, cytokines discussed above (e.g., TNF-α, IL-6) have been known as a fundamental part of cholangiocyte SASP (105).

Other than supportive functions in hepatocellular proliferation, activated cholangiocytes conduct a ‘self-rescuing’ program to sustain their own proliferation and survival. During this ‘self-rescuing’ procedure, cholangiocellular proliferation is initiated not only by genetic/epigenetic alterations but also by autocrine/paracrine cytokines. As described previously, IL-8 levels increase in PSC patients’ bile. In addition, IL-8 caused cell proliferation when added to primary human cholangiocyte cultures (28). The bile component TC protects cholangiocytes against injury. In mouse BDL models, TC administration can enhance VEGF-A and VEGF-C, which are key regulators of biliary proliferation during cholestasis (159, 160). Gigliozzi et al. demonstrated that cholangiocytes secrete NGF, which stimulated the proliferation of cholangiocytes *via* protein kinase B (AKT)- and ERK1/2-dependent mechanisms. *In vivo*, NGF neutralization decreased the proliferative capacity of BECs in post-BDL rats (47). More interestingly, we reported that the secretion of CCL-2 by injured cholangiocytes attracts monocytes, which in turn upregulate integrin-β6 and favor cholangiocyte proliferation. This study proposed a novel concept regarding cholangiocyte-associated cellular crosstalk during liver injury (112, 161). Taken together, bile duct repair is driven by stimulatory and inhibitory, autocrine or paracrine secretory factors originating from cholangiocytes. Promisingly, variable cells may be involved in the complex regulation of such regenerative processes.

### Inflammation

Inflammation is a fundamental orchestrator of BEC response to liver injury. As discussed above, inflammatory factors effectively influence the cholangiocyte secretory programs. In turn, cholangiocytes with active secretory phenotypes regulate immune cell accumulation and polarization.

Cholangiocytes are capable of sensing exogenous stimuli, including PAMPs and DAMPs, *via* TLRs and the downstream signal pathways. Upon sensing these stimuli, signaling cascades mainly involving NF-κB, mitogen-activated protein kinase (MAPK) and inflammasome, are rapidly activated (102, 111). Consequently, a broad spectrum of proinflammatory cytokines (e.g., IL-1β, IL-8, IL-6, MCP-1, TNF-α, INF-γ and TGF-β) and chemokines (e.g., CXCL-1, -8 and -16), is released by cholangiocytes (2, 162, 163). Investigations of the liver immune landscape revealed that, the recruited leukocytes are the leading responders in the immune response towards bile duct alterations (2, 164). The first single-cell analysis of liver samples from PSC patients indicates a strong dynamism of T cells, among which naive CD4^+^ T cells are prone to develop into T-helper (Th) 17 cells (165). Th17 cell accumulation has also been observed in the liver biopsies of PBC patients, specifically around the activated or injured intrahepatic BECs (75, 166). Reactive cholangiocytes regulate Th17 cell differentiation by IL-6 and IL-1β (167). In addition, fractalkine/CX3CL1 and CXCL1 are released by reactive cholangiocytes, which further recruit monocytes and T cells (109, 131, 168, 169). In response to biliary injury, injured or senescent cholangiocytes dramatically release TNF-α and IL-6 (105, 134). TNF-α not only activates naïve and effector T cells, but also induces apoptosis of highly activated effector T cells, further determining the scale of the pathogenic or protective conventional T-cell pool (170). Meanwhile, IL-6 is not only a key player in regulating the Th17/Treg balance, but also exerts paracrine functions to promote terminal differentiation of B cells and their subsequent secretion of immunoglobulins (171, 172).

Another important part of the liver’s innate immunity is the hepatic myeloid cells, which execute crucial roles in either driving liver injury or repairing hepatic malfunction in liver diseases, such as cholangiopathies (161, 173, 174). We have revealed the cholangiocyte-monocyte crosstalk using an acute biliary cell injury mouse model. We found that the injured cholangiocytes can promote the accumulation of CCR2^+^ monocyte-derived macrophages (MoMFs) and alter bile acid metabolism, while the MoMFs provide important factors for cholangiocytes to proliferate and restore biliary function (112). Furthermore, we have learned from the liver samples of PSC patients and th*e ex vivo* experiments that, secretion of CCL-20 and CCL-2 from human primary cholangiocytes favors monocyte infiltration (175). Additionally, Mip-3a/CCL-20 can be released by the activated cholangiocytes to induce the chemoattraction of immature dendritic cells by its binding to CC chemokine receptor 6 (CCR6) (73). To sum up, cholangiokines play a crucial role in hepatic immunomodulation. However, a more precise understanding of cholangiocyte-driven inflammation is necessary.

### Fibrosis

In response to an injury, DR is driven by cholangiocyte proliferation and their secretome, participating in the complex regulation of portal inflammation and fibrogenesis (14). Inflammation generates signals that attract liver mesenchymal cells to bile ducts and portal areas. This process is considered to be the primary stage of biliary or portal fibrosis. In this context, interaction between reactive ductular cells and myofibroblast cells, so-called epithelial–mesenchymal crosstalk, is a constant key modulator in liver fibrogenesis, the process of which also involves several profibrogenic factors (e.g., IL-6, TGF-β1/2, CCL-2 and PDGF-B) (76, 94, 95).

During biliary fibrosis, proliferating BECs represent a predominant source of the profibrogenic connective tissue growth factor (CTGF) besides HSCs (176, 177). According to a recent study, reactive cholangiocytes secrete TGF-β depending on the Mothers against decapentaplegic homolog 3 (SMAD3) and lysine Acetyltransferases 2A (KAT2A). Pharmacological inhibition of Kat2a protein or cholangiocyte-selective deletion of *Kat2a* gene was protective in mouse models of biliary fibrosis (178). BECs can regulate the proliferation and myofibroblastic trans-differentiation of HSCs to provoke the portal fibrosis by the CCL-2-based paracrine (94). TGF-β1 and TGF-β2 were found upregulated in cholangiocytes during chronic liver diseases, suggesting their implication in biliary hyperplasia and fibrogenesis (76). Likewise, IL-8 secreted by the activated cholangiocytes can stimulate the production of profibrotic genes, suggesting that IL-8 may be involved in the pathogenesis of cholangiopathies (28). Grappone et al. suggested that PDGF-B chains can be produced by cholangiocytes during chronic cholestasis (179). Recently, Moncsek et al. disclosed that senescent cholangiocytes promoted the activation of quiescent mesenchymal cells in a PDGF-dependent manner (180). Another latest study has demonstrated that biliary NF-κB-inducing kinase (NIK) could trigger DR. While the ablation of NIK significantly decreased the expression of *Il-1β*, *Il-4*, *Il-6*, *iNos*, *Tnfα*, *Mcp1* and *Tgfb1*, thereby attenuating liver fibrosis (25). What’s more, Liu et al. reported that cholangiocyte-derived exosomal H19 plays a critical role in the progression of cholestatic liver fibrosis by promoting the differentiation and activation of HSCs (181). Integrin αvβ6 acts as not only a crucial mediator but also a therapeutic target in liver fibrosis (182, 183). Moreover, genetic suppression of *Itgb6* (a gene encoding integrin αvβ6) in the mouse models of biliary injury is therapeutically relevant to the attenuation of DR and biliary fibrosis (184, 185). Pi et al. have revealed that CTGF and integrin αvβ6 regulate biliary cell activation and fibrosis, probably through the secretion of fibronectin and TGF-β1 (176). In conclusion, activated cholangiocytes and their cholangiokines might be promising therapeutic targets for ameliorating liver fibrosis.

### Carcinogenesis

Primary liver cancers, including hepatocellular carcinoma (HCC) and CCA are a tremendous burden to global health, but their pathomechanisms are only partially understood (137, 186). From a short-term perspective, cholangiokines contribute to hyperplasia, inflammation and fibrogenesis of the hepatic portal areas. In the long run, cholangiokines may eventually fuel the malignant transformation of hepatic cells through continuous autocrine and paracrine stimulation.

IL-6 has been determined by several studies as not only a key driver but also a promising therapeutic target for liver cancers (187–189). IL-6 levels are highly presented in the serum and bile of CCA patients and culture medium of CCA cell lines (190). Recent studies concluded that HCC and intrahepatic CCA (iCCA) are significantly driven by IL-6 and its associated inflammatory processes (191, 192). IL-6 promotes the survival of transformed cholangiocytes through different pathways. In particular, the IL-6-activated p38 pathway determines cell proliferation by mediating p21^WAF1/CIP1^ and p44/p42 MAPK (193). Even more intriguingly, single-cell analysis of iCCA patient specimens showed that CCA-derived exosomal miR-9-5p elicited a high secretory possibility of IL-6 in cancer-associated fibroblasts to promote tumor progression, suggesting broader roles of cholangiocyte- derived IL-6 in the tumor microenvironment (TME) (194).

EGF administration can provoke CCA progression by triggering epithelial-mesenchymal transition (EMT). In addition, the upregulation of TGF-α favors the proliferative levels of HCC cells (195). EGF and TGF-α regulate cell proliferation and differentiation by binding EGFR (196). Earlier studies also revealed a positive correlation between EGFR inhibition and HCC suppression (197, 198). Inoue et al. characterized that blocking EGFR by vandetanib in liver cancer models yielded a significantly reduced tumor vessel density and tumor growth, while enhancing tumor cell apoptosis and survival prolongation with reduced number of intrahepatic metastases (199). Moreover, it has been well elucidated that hepatic myofibroblasts promote malignancy progression in CCA patients through their HB-EGF-induced activation (156, 200), which is consistent with the fact that myofibroblasts are also prone to trigger the cholangiocyte-secreted PDGF-B (201).

TGF-β and its related signaling cascades play a central role in inflammation, fibrogenesis and immunomodulation in the TME of liver cancers (202, 203). A recent study indicated a positive feedback loop of TGF-β and LIN28B in CCA metastasis (204). TGF-β has also been found to promote the progression of CCA and HCC by interacting with non-coding RNAs (205–208). More strikingly, TGF-β exerts immunoregulatory functions in HCC, mainly *via* suppressing T cells (202, 209). Interestingly, the blockade of TGF-β-induced activated dendritic cells enhances the lethal effects of T cells in CCA (210). Thus, TGF-β potentially disturbs immunotherapies in liver cancers, which makes it a promising target to attenuate immunotherapy resistance. Besides, TGF-β was also found to regulate monocyte/macrophages in liver cancers. Yan et al. reported that TGF-β fosters the expression of T cell immunoglobulin domain and mucin domain-3 (TIM-3/CD366) on monocytes, which augments the infiltration of tumor-associated macrophages in HCC (211). Ning et al. demonstrated that the induction of imbalanced TGF-β1/BMP-7 pathways in HCC cells could significantly reinforce the aggressiveness and stemness of HCC cells (212).

Novel observations indicate that VEGF is a master factor in lymphangiogenesis and the immune response to cholangiocarcinoma (84). The secretion of VEGFs, angiopoietin-1/2, PDGF and TGF-β from tumor cells or other cell types robustly modulate the TME, which is a critical component of tumor biology (213). The VEGF-A secretion by CCA cells can be mediated by other factors including IGF-1, its receptor IGFR as well as the estrogen receptor (ER) family (214, 215). Furthermore, estrogens induce the proliferation of CCA cells by VEGF/VEGFR2 mediation (216). VEGF-A, on the other hand, induces cholangiokines, including matrix metalloproteinase (MMP)-7 and -9, from CCA cells, which contribute to the significant remodeling of extracellular matrix (ECM) and the extensive tumor metastasis (217).

Notably, TNF-α plays contradictory roles in liver cancers. Commonly known as a participant in maintaining homeostasis of cancer immunobiology, TNF-α unveils its ‘dark side’ to provoke chronic inflammation, EMT and angiogenesis, which may fuel the aggressiveness of cancers (218). Interestingly, high-dose administration of TNF-α inhibits neovascularization in mice, whereas low –dosed TNF-α promotes angiogenesis by increasing the expression of VEGF, VEGFR, IL-8 and basic FGF (219). Another study underlined that TNF-α strengthened the migration behaviors of CCA cells by upregulating their EMT markers, including ZEB2, vimentin and S100A4. Moreover, TNF-α has been shown to induce *TGFB* overexpression, which eventually promotes cancer cells to migrate (220). Yuan et al. described a novel phenomenon that TNFs favor cholangiocellular proliferation, differentiation and transformation due to the induced chronic mitochondrial dysfunction and the accumulation of reactive oxygen species (ROS). This finding enriches the research directions of TNF-α meditation in CCA (221). Even though cholangiokines can hardly be concluded as a robust oncogenic secretome based on our current knowledge, various tumor-promoting cytokines secreted by cholangiocytes have been evidenced to regulate TME.

## Conclusions and future perspectives

Although the quantitative contribution of cholangiocytes to the total liver mass and the hepatic secretome appears modest, cholangiocytes play essential roles in a vast array of disease-related mechanisms and shape the portal area microenvironment. Sensitized by various injuries, stimuli or immune disturbances, cholangiocytes release cholangiokines, which broadly participate in liver immunology, inflammation, fibrogenesis and malignant transformation. Particularly, cholangiokines are gaining recognition for their involvement in cholangiopathies and primary liver cancers. Of note, better characterization of the cholangiokines may provide an in-depth understanding of cholangiocyte-driven pathophysiological processes. Nonetheless, the paracrine and autocrine nature of cholangiokines poses some technical challenges, as their functions need to be interpreted in the spatiotemporal context of the hepatic microenvironment. Even though the practicability of cholangiokines as diagnostic/prognostic markers is still hidden in fog, emerging biotechnics can incarnate wind to achieve it. Recently, several novel approaches, such as multiplex immunostaining, imaging mass cytometry and spatially resolved single-cell sequencing, have emerged for *in situ* liver studies, which shed light on differential spatial heterogeneity of the hepatic parenchymal and immune cells (164, 222–224). Furthermore, by tying up the single-cell spatial or newly developed single-cell Stereo-sequencing methods (225), pathomechanisms of cholangiokines associated with time phases, zonation and functionality are anticipated to be soon and decently determined.

## Author contributions

HL conceived the topic. XC and HL drafted the manuscript and prepared the figures. XC, FT, AG, and HL revised the manuscript. All authors have approved the published version of the manuscript. All authors contributed to the article and approved the submitted version.
